# Single Anesthetic Approach to Diagnosis, Staging and Treatment of Lung Cancer

**DOI:** 10.3390/cancers18182928

**Published:** 2026-09-10

**Authors:** Matthew Aizpuru, Jackson Wittenberg, Janani Reisenauer

**Affiliations:** Division of Thoracic Surgery, Department of Surgery, Mayo Clinic, 200 First St. SW, Rochester, MN 55906, USA

**Keywords:** single anesthetic event, single stage, lung cancer, adenocarcinoma, ss-RAB, robotic bronchoscopy

## Abstract

The conventional workup for suspected early-stage lung cancer requires multiple visits, anesthetics, and procedures. Single anesthetic event lung cancer surgery integrates shape-sensing robotic bronchoscopy, cone-beam CT, rapid on-site cytologic evaluation (ROSE), endobronchial ultrasound staging, lesion localization, and minimally invasive resection into one operative encounter. In this narrative review, we describe the technical components of this pathway, summarize the supporting literature, and offer expert, institution-based troubleshooting guidance for common intraoperative dilemmas. Reported diagnostic yields for shape-sensing robotic bronchoscopy range from 80 to 96%, with approximately 90% concordance between ROSE and final pathology. Published single-institution series report reductions in time from detection to resection of 15–51 days, cost savings of approximately $3000–$10,000, and perioperative outcomes (length of stay 1.8–3.6 days; complication rates comparable to traditional pathways) similar to staged care. The supporting evidence is retrospective and derived from small, single-institution, high-volume referral cohorts; no prospective comparative trials or long-term survival data yet exist. Single anesthetic event lung cancer surgery is therefore best regarded as an emerging, resource-intensive care pathway for carefully selected patients at experienced centers rather than an established standard of care, pending prospective, multicenter validation.

## Simple Summary

Single anesthetic event lung cancer surgery is an innovative approach involving the integration of several technological advancements in minimally invasive thoracic surgery and bronchoscopy to streamline the diagnosis, staging and treatment of early-stage non-small-cell lung cancer. By combining shape-sensing robotic bronchoscopy, endobronchial ultrasound, rapid on-site cytology, lesion localization and minimally invasive surgical resection into a single anesthetic event, time from detection to resection may be significantly improved.

## 1. Introduction

Single anesthetic event lung cancer surgery is the culmination of years of technological advancement resulting in a minimally invasive bronchoscopic and surgical technique for the complete diagnosis, staging and treatment of lung cancer in a single episode of care. Lung cancer remains the leading cause of cancer-related death with nearly 125,000 deaths in the United States in 2025 [1]. As a result of smoking cessation, lung cancer screening, improved systemic therapy and improved surgical treatment, lung cancer survival has improved significantly over the last two decades. Overall median survival following diagnosis of lung adenocarcinoma has improved from 8.5 months to 20.7 months from the year 2000 to the year 2020 [2]. Much of this improved survival is driven by remarkable advancements in the diagnosis and treatment of early-stage disease, where 3-year overall survival has improved from 49.5% to 78.8% in that same interval [2]. The advent of minimally invasive surgery, early recovery after surgery (ERAS) pathways, improved perioperative care, better staging, and the introduction of quality metrics in surgery all likely contributed to these improvements. Despite the advances that have been made, there are still opportunities to reduce the invasiveness of treatments, while conferring the maximum quality and quantity of life to patients with lung cancer.

Each element of the diagnosis, staging and treatment of lung cancer has improved remarkably over the last decades. Lung cancer screening, more frequent use of computed tomography and increased use of FDG PET/CT have improved early diagnosis. Better imaging, endobronchial ultrasound fine-needle aspiration and improved understanding of mediastinal lymph node assessment have improved staging. Surgery has evolved from open thoracotomy to minimally invasive approaches such as video-assisted thoracoscopy (VATS), robotic-assisted thoracoscopy (RATS). The standard of care has also changed from lobectomy alone to including sublobar resection for appropriately selected small peripheral tumors [3,4]. Patients with advanced disease now have access to improved systemic treatment options with the addition of immunotherapy and targeted therapies [5,6,7,8].

An unforeseen consequence of these advances is an increasingly complex and burdensome diagnostic process involving numerous appointments and tests, which come at a significant cost to patients and their families. Patients often undergo multiple CT scans, specialized imaging tests, and invasive procedures such as transthoracic needle biopsy, endobronchial ultrasound or cervical mediastinoscopy before definitive treatment is undertaken. For some patients, the time, travel, missed work and financial burden is too much for them to receive the recommended workup and treatment.

To streamline the diagnosis, staging and treatment of lung cancer, specialized centers have begun offering what is termed single anesthetic event lung cancer surgery, wherein patients undergo definitive diagnosis, staging and treatment during a single procedure. This integrated process eliminates the need for repeated appointments, anesthetics, and procedures. In this narrative review, we describe the technical components of single anesthetic event lung cancer surgery, the data around each part of the procedure, the supporting data for the approach, and share expert insights for these cases. We emphasize throughout this review that single anesthetic event lung cancer surgery currently represents an emerging, institution-driven care pathway for carefully selected patients in appropriately resourced and experienced centers, rather than an established standard of care for early-stage non-small-cell lung cancer. The literature included in this review is based on a search of PubMed/MEDLINE with search terms including “single anesthetic event,” “robotic bronchoscopy,” and “rapid on-site evaluation,” for articles published between 1 January 2020 and 31 July 2026, supplemented by expert knowledge of the field and hand-searching of reference lists; articles were included if relevant to a review of the subject. As this is a narrative rather than a systematic review, this search strategy was not intended to be exhaustive or to follow a PRISMA framework. Quantitative data presented in this review have not been systematically collected or analyzed and therefore should not be interpreted as pooled estimates.

## 2. Background

The standard approach to the diagnosis, staging and treatment of early-stage non-small-cell lung cancer involves imaging, preoperative biopsy in cases where lobectomy would be considered, and invasive mediastinal staging for larger and more central tumors [9]. Patients often undergo multiple visits, tests, and procedures to establish the diagnosis and stage their disease. Completion of this workup takes time, and multiple studies have demonstrated that delays in diagnosis and treatment result in lung cancer upstaging [10,11,12,13]. The aim of single anesthetic event lung cancer surgery is to streamline this process into a single operative room event, where all necessary steps can be performed under the same anesthetic, resulting in more timely care. It should be emphasized that the evidence base supporting this approach remains almost entirely retrospective and single-institutional; this qualification is discussed in detail below, but applies to the interpretation of the entire review.

Typical indications for preoperative biopsy include scenarios where neoadjuvant systemic therapy is being considered (tumor greater than or equal to 4 cm, nodal involvement suspected), or where the nodule is >2.0 cm or central, and a lobectomy would be required for resection [9,14]. In modern practice, guidelines specifically recommend against performing diagnostic lobectomy, bilobectomy or pneumonectomy, unless attempts at biopsy have been exhausted [15]. Biopsy has traditionally been performed via transthoracic needle puncture; however, this has been associated with significant risk of pneumothorax [16]. There have been significant technological advancements in this space. Flexible bronchoscopy, endobronchial ultrasound (EBUS), endobronchial ultrasound with transbronchial needle aspiration (EBUS-TBNA), electromagnetic navigational bronchoscopy, shape-sensing robotic bronchoscopy, and cryobiopsy have greatly improved the ability to complete diagnosis and staging of lung cancer through the natural orifice of the mouth and airway [17].

Because many screen-detected pulmonary nodules are small and peripheral, conventional flexible bronchoscopy is limited by difficulty in reaching distal nodules. Robotic-assisted bronchoscopy allows access to the distal airway for lesion sampling. Robotic bronchoscopy platforms such as the Ion Endoluminal System (Intuitive Surgical), MONARCH Platform (Johnson and Johnson), and The Galaxy System (Noah Medical) robotic bronchoscopy platforms have improved peripheral reach, and diagnostic accuracy of bronchoscopy [18,19]. This technology creates the foundation for single anesthetic event workflows.

## 3. Diagnostic Methods

Central to the concept of single anesthetic event lung cancer surgery is the need for immediate and accurate diagnosis and staging in the hybrid operating room. As each step in the procedure builds on the findings of the previous step, accurate diagnostics are critical to safe patient care. For example, the surgeon would want to confirm malignancy, before proceeding with EBUS, and again confirm the cancer stage before proceeding with resection. In the case of bronchoscopic biopsy, the two key issues are the effective sampling with the needle biopsy, and the reliability of the on-site pathology results.

The choice of bronchoscopic platform may impact the diagnostic accuracy, and therefore the ability of the surgeon to integrate all steps into a single procedure. Traditional bronchoscopy with transbronchial biopsy cannot reliably access small, peripheral lesions, limiting its utility in single anesthetic event surgery. There are several navigational bronchoscopy technologies available, and a comparison of them is beyond the scope of this review article [20]. Robotic-assisted bronchoscopy utilizes shape-sensing technology (Ion), real-time tomosynthesis (Galaxy), or electromagnetic assistance (Monarch) for localization. Studies comparing electromagnetic navigation bronchoscopy with the shape-sensing robotic-assisted bronchoscopy platforms have not definitively demonstrated a difference in diagnostic yield, but access to small and peripheral nodules is improved with shape-sensing robotic-assisted bronchoscopy [21,22,23,24]. Single anesthetic event lung cancer surgery is typically performed using a reliable bronchoscopic system, and in many centers, shape-sensing robotic-assisted bronchoscopy. Robotic-assisted bronchoscopy has significant catheter stability, which allows for excellent access to small nodules and upper lobe lesions, which may be challenging with alternative techniques. The diagnostic yield for shape-sensing robotic-assisted bronchoscopy cases has been shown to be 80–96% in large series [25,26,27,28,29]. Robotic-assisted bronchoscopy has an excellent safety profile, with overall complication rates around 3.0% and pneumothorax rates around 2.0% [29,30]. When compared to transthoracic needle biopsy, robotic-assisted bronchoscopy with biopsy has a similar diagnostic yield with significantly reduced complication rates [31,32]. A recent randomized control trial published in the New England Journal of Medicine compared navigational bronchoscopy with biopsy to TTNB, and found similar diagnostic yield and reduced complications for navigational bronchoscopy [16].

**Figure 1:**
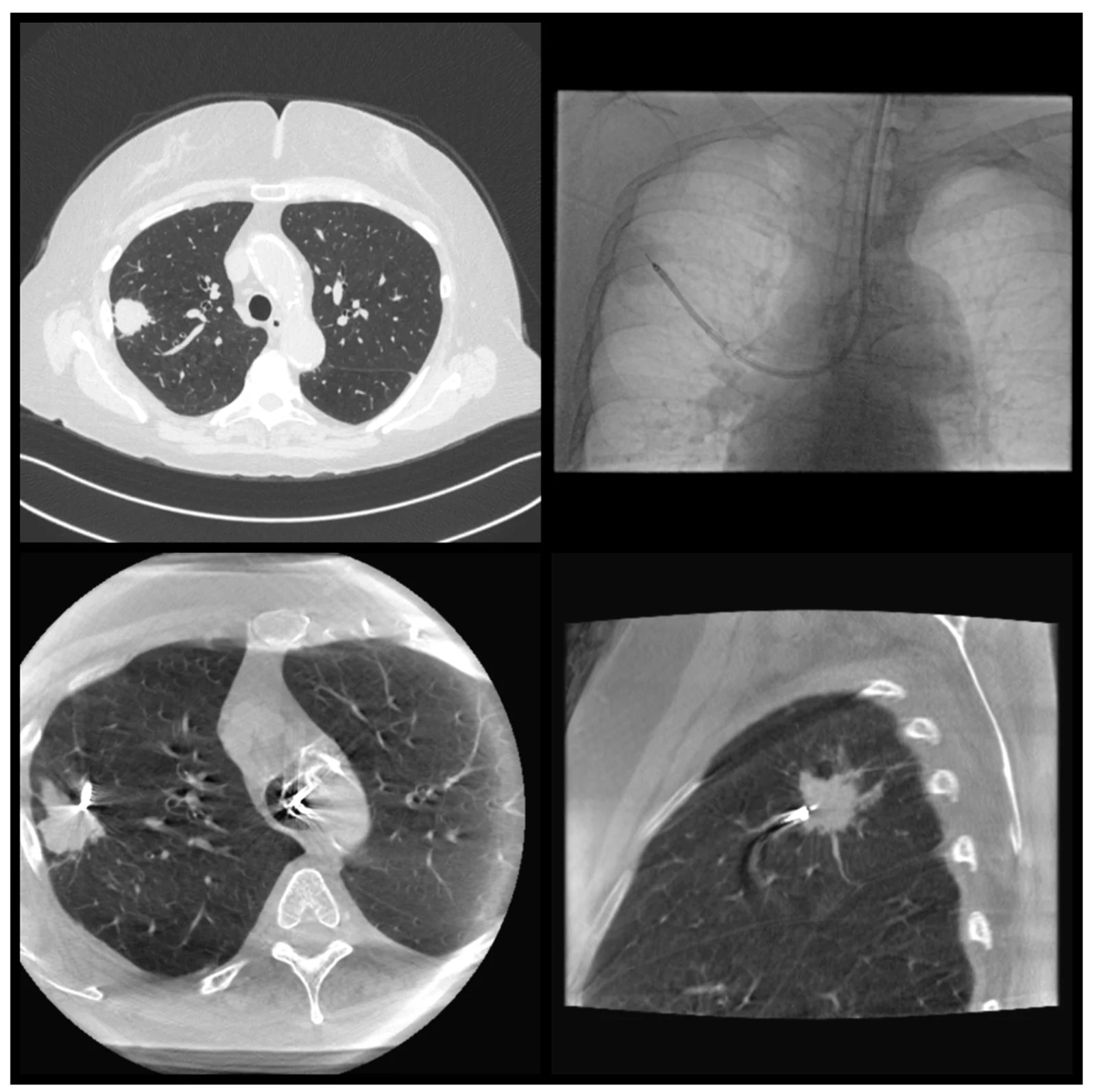
Example of the use of cone-beam CT to confirm the catheter position with “tool-in-lesion” for this right upper lobe nodule.

The addition of mobile and/or fixed cone-beam CT can further improve the diagnostic yield and accuracy of robotic-assisted bronchoscopy. This allows the surgeon to position the catheter in the expected location based on shape sensing, and confirm the anatomy with an on-table cone-beam CT scan (Figure 1). A recent multicenter prospective study, the CONFIRM study, examined the combination of shape-sensing robotic-assisted bronchoscopy with mobile cone-beam CT and found the diagnostic yield to be 89%, with a sensitivity for malignancy of 92% [18].

Cryobiopsy can further improve the diagnostic yield of robotic-assisted bronchoscopy. Shape-sensing robotic-assisted bronchoscopy platforms typically facilitate the use of 1.1 mm and 1.7 mm cryobiopsy probes. The FROSTBITE-2 trial compared 1.1 mm cryobiopsy to 2.0 mm forceps biopsy and found higher diagnostic yield using cryobiopsy [33]. Specific to shape-sensing robotic bronchoscopy, the diagnostic yield of cryobiopsy has been reported to be 92% and cryobiopsy is shown to be an independent predictor of diagnosis [25,34].

Once tissue has been obtained from the primary lesion, cytopathologic evaluation with rapid on-site evaluation (ROSE) is necessary to make the diagnosis. ROSE is essential to the single anesthetic event program because it allows immediate assessment of specimen adequacy and preliminary diagnosis during bronchoscopic sampling. The surgeon must work closely with the cytotechnologist to identify and diagnose adequate samples intraoperatively in real time. When using ROSE in the context of robotic bronchoscopy FNA biopsies of primary lesions, the concordance of ROSE and final pathology is approximately 90% [35,36]. Inability to confirm malignancy at the time of robotic bronchoscopy is one of the main barriers to a successful single anesthetic event lung cancer surgery. For this reason, extensive preoperative counseling via a shared decision-making approach with the patient is recommended. A plan as to how the team will proceed if there is ongoing diagnostic uncertainty should be established and documented prior to entering the operating room. Published series of single anesthetic event lung cancer surgery report high rates of concordance between intraoperative rapid on-site evaluation findings and final pathologic diagnoses approaching 90%, with acceptably low rates of benign resection [26,37,38]. While a 90% concordance rate is favorable from a diagnostic standpoint, it may be viewed as inadequate when the implication is a potential benign resection. A false-positive ROSE interpretation, in which benign tissue is read as malignant, risks an unnecessary resection, while a false-negative interpretation risks inappropriately deferring a needed intervention or proceeding with an incomplete oncologic operation. The degree of diagnostic certainty required to proceed is increased with the extent of planned resection. For example, a surgeon may be comfortable performing a small wedge without a definitive diagnosis, while a right lower lobectomy should have a much higher threshold of diagnostic certainty. This issue is discussed further, including specific troubleshooting strategies, in Section 7.1.

Once a cancer diagnosis has been established, invasive mediastinal staging is frequently necessary prior to surgical resection. For single anesthetic event lung cancer surgery, this is typically performed as an EBUS-TBNA. ROSE adds value to EBUS-TBNA by reducing the number of needle passes necessary to obtain adequate specimens [39]. For single anesthetic event lung cancer surgery, ROSE is necessary to stage the mediastinum prior to proceeding with resection. ROSE during EBUS-TBNA nodal staging has been shown to have a sensitivity of 90% and specificity of 97% [40].

Overall, a single anesthetic event approach to diagnosis, staging and resection requires concordance between preliminary cytology and final pathology results [35]. This means that the surgeon must get adequate tissue samples from the primary tumor and any mediastinal lymph nodes that are sampled. The cytopathologist must be able to provide accurate interpretations of the slides, and those interpretations must be concordant with final pathology results with an acceptable margin of uncertainty.

## 4. Patient Selection

There are several scenarios in which a single anesthetic event lung cancer surgery is a useful strategy (Table 1). Potential candidates for the single anesthetic event approach should by definition also be candidates for shape-sensing robotic-assisted bronchoscopy. In most published series, target nodules have ranged from approximately 8 mm to 30 mm in size, peripheral or mid lung in location, and pleural- or fissure-based [41]. Ideal candidates for single anesthetic event lung cancer surgery are patients who meet criteria for shape-sensing robotic bronchoscopy, are otherwise healthy, with a suspicious nodule amenable to sublobar or lobar resection, and where some combination of at least two of biopsy, localization, mediastinal staging, and resection is needed. Factors favoring a single anesthetic event approach include a high probability of malignancy, clinical T1-T2aN0M0 disease, no prior tissue diagnosis, BMI < 38 to allow for appropriate positioning and ability to perform cone-beam CT spin, and a likelihood of completing the resection minimally invasively [37,41,42].

Relative contraindications to single anesthetic event lung cancer surgery include intermediate pretest probability of malignancy, very small nodules, small predominantly ground-glass nodules, central tumors or those abutting major vascular structures or airways, patients with difficult airways, and patients with significant medical comorbidities [41,42]. These features should not be viewed as automatically excluding a patient from the combined pathway; rather, they warrant more individualized decision-making that weighs pretest probability of malignancy, solid versus subsolid nodule morphology, lesion location, anticipated extent of resection (wedge/segmentectomy versus lobectomy), the need for invasive mediastinal staging, the patient’s pulmonary reserve and comorbidities, and most importantly, a plan for how the team will proceed if intraoperative diagnosis remains uncertain (see Table 1 and Section 7.1). Body mass index (BMI) has been demonstrated to correlate to degree of procedural atelectasis, which can impact CT registration for robotic bronchoscopy [43,44]. Morbid obesity with BMI ≥ 38 may represent a relative contraindication to single anesthetic event surgery for this reason.

Absolute contraindications include locally advanced tumors such as those with N2/3 nodal or metastatic disease [9], medically inoperable patients, patients who cannot tolerate general anesthesia or single lung ventilation.

## 5. Operative Steps

### 5.1. Preoperative Assessment and Planning

The robotic-assisted bronchoscopy should be conducted in concordance with guidelines [41]. The thin-slice CT chest is uploaded to the robotic platform’s planning software [45]. The planning laptop is then used to segment the CT scan into a 3-dimensional model. The software generates a pathway to the nodule of interest. Though not explicitly recommended in guidelines, creating two pathways to the nodule should be considered, as this can help overcome some degree of misregistration [45]. Planning can be performed the day prior to the surgery, which can help avoid unexpected intraoperative delays [43]. Anecdotally, while the most direct pathway is usually optimal, perfectly straight trajectories can occasionally hinder oblique needle passage through the bronchial wall; in such cases, a more angled alternative is preferred. Pathways requiring significant bending of the catheter as measured by bend radius close to or less than 15 mm are often troublesome.

### 5.2. Operating Room Setup

Single anesthetic event lung cancer surgery is performed in a hybrid OR with on-table fluoroscopy and cone-beam CT capability. The same room must facilitate the bronchoscopic platform to be utilized, and video or robotic-assisted thoracoscopy. After general anesthesia is induced with at least an 8.5 mm diameter endotracheal tube, the patient must be precisely positioned to allow for free movement of the cone-beam CT gantry [41]. Using a double lumen tube directly, and positioning lateral from the outset has been described as an alternative to improve operative workflow [46].

### 5.3. Bronchoscopy

Flexible bronchoscopy can be performed, and then the ss-RAB docked. Clearance of the cone-beam CT should be performed with a “practice spin” prior to docking the robot to ensure appropriate isometric configuration of the C-arm and appropriate positioning of the robot. As a matter of institutional practice rather than published guideline, the positive end expiratory pressure is set to 10 mmHg for upper lobe lesions, and 12 mmHg for lower lobe lesions to achieve good lung inflation for registration. Other institutional protocols exist for avoidance of atelectasis [43]. Avoidance of high FiO2 is preferred to further reduce atelectasis as oxygen-rich environments can lead to alveolar collapse, making navigation more difficult [41]. Using a combination of the vision probe and the virtual pathway, the robotic bronchoscope is directed towards the target lesion. The ideal position of the catheter is within 3 cm of the target, with minimal bend radius and against an airway wall [41]. Confirmatory imaging, whether radial EBUS or cone-beam CT demonstrating tool-in-lesion, should be performed with a positive pressure breath hold during spin [41]. Multiple passes of the nodule are taken with the needle of choice, at provider discretion. If the ROSE is non-diagnostic, then assessment of the catheter position should be performed. Additional biopsies with forceps or a 1.1 mm flexible cryoprobe can be performed [41]. Once diagnostic material has been identified, the procedure can proceed as planned.

### 5.4. Marking of the Lesion for Sublobar Resection

Several options exist for marking a lesion for sublobar resection. The most commonly employed techniques include indocyanine green coil placement and injection of ICG and/or methylene blue [47,48,49,50]. Confirming the position of the catheter is critical prior to marking the lesion. Fluoroscopy and cone-beam CT can be utilized to ensure accurate lesion localization.

### 5.5. Mediastinal Staging

Following tissue diagnosis, invasive mediastinal staging can be performed if needed. Once EBUS-TBNA is complete, a flexible bronchoscopy should be performed to suction any bloody secretions prior to proceeding with double lumen endotracheal intubation.

### 5.6. Minimally Invasive Pulmonary Resection with Lymphadenectomy

Following completion of diagnosis and staging, surgical resection can be undertaken. The single lumen endotracheal tube is exchanged for an appropriate-size double lumen endotracheal tube. The patient is positioned in lateral decubitus position. On a fluoroscopy bed, this can present a unique challenge. We utilize chest rolls, an axillary roll, and an arm board to secure the patient. To simulate flexion of an operating room table, we fashion a bump out of two rolled blankets, a gel pad, and a bed sheet. This is then placed under the patient at the level of the xiphoid process. The axillary role helps ensure that the patient is adequately supported counter to this bump. Once positioning is satisfactory, minimally invasive resection can be completed in the standard fashion. This positioning technique reflects our own institutional practice rather than a formally validated or comparative standard; alternative positioning strategies may be equally effective and have not been directly compared.

## 6. Outcomes of Single Anesthetic Event Lung Cancer Surgery

Data supporting single anesthetic event lung cancer surgery is largely single institution and retrospective. A summary of the most important outcomes from multiple studies is displayed in Table 2.

The impetus for establishing a single anesthetic event lung cancer surgery program is to reduce the time between detection and treatment for early-stage disease. Significant improvements in time from detection to resection have been reported, ranging from 15 to 51 days and roughly a 30% reduction in wait time [37,51,52]. While direct survival benefits of single anesthetic event lung cancer surgery have not been demonstrated, delayed treatment has been associated with significant reductions in survival [54].

The reduced burden of single anesthetic event lung cancer surgery programs is best considered along two related but distinct dimensions: patient-centered outcomes and health-system outcomes. Total cost savings ranging from $3000 to $10,000 have been described [37,51]. These estimates are derived from relatively small, single-institution cohorts, and may not be generalizable. Published economic analyses have also focused primarily on savings from consolidating multiple procedures into one, while the opportunity cost of occupying a hybrid operating room for a prolonged combined case, which may limit throughput for other patients, has not been well-characterized and warrants consideration in future cost analyses. In addition to direct costs to the hospital and patient, indirect costs may be improved as well. Patients can continue to work up until the day of surgery, and require less anesthetics, missed workdays and likely appointments. Psychological burdens may be reduced as well, though studies on patient reported outcomes are lacking. Whether patients themselves prefer the single anesthetic event approach remains an open and important question. Some patients will value avoiding multiple procedures and visits, while others may instead prefer to receive a definitive diagnosis, and understand their options before undergoing lung resection. Future studies should incorporate patient-reported outcomes, including decisional regret, satisfaction and anxiety.

Perioperative outcomes appear to be similar between single anesthetic event and traditional care pathways. Length of stay has been reported from 1.8 to 3.6 days [26,51,52,53]. Rates of pneumothorax, bleeding, and perioperative mortality have been described as similar between the single anesthetic event approach and traditional pathways [37,42,51].

Critics of the single anesthetic event concept cite increased operative times as a main limitation of the technique. It is true that operative times for a single anesthetic event lung cancer surgery typically exceed those of standard pulmonary resections. Operative times of greater than 3 h are commonly reported [26,37,42,53]. Due to consolidation of anesthesia and turnover times by combining procedures, overall time in the procedural suite, however, has been shown to be reduced significantly in single anesthetic event programs, with total time in suite reduced by between 54 and 231 min [37,42,53].

One additional potential benefit of single anesthetic event lung cancer surgery is the avoidance of benign resection. For example, patients who would have been marked by CT guidance on the day of surgery may undergo bronchoscopic biopsy, followed by bronchoscopic marking and resection. In these types of scenarios, rates of benign resection have been reported to be reduced from 21.9% to 7.6% [42].

## 7. Troubleshooting

When each step of the single anesthetic event pathway proceeds without incident, it provides an efficient and effective pathway for patient care. In many cases, however, the surgeon faces unforeseen scenarios and difficult judgement calls, which can add to the complexity of the overall care plan for the patient. Many of the intraoperative decisions in these cases are not evidence-based; throughout this section we explicitly distinguish evidence-supported recommendations (cited accordingly) from expert consensus and our own institutional practice or opinion, which we identify as such.

### 7.1. Rose Is Indeterminate

Perhaps the most difficult situation the surgeon faces during single anesthetic event lung cancer surgery is indeterminate ROSE results despite multiple biopsies. This situation has been reported to occur in 8.3–25.6% of cases [37,38]. In this scenario, the first step is to ensure adequate sampling, whether that requires repositioning of the catheter, or use of an alternative targeting pathway. Tool-in-lesion should be demonstrated on imaging when feasible. The addition of cryobiopsy can improve diagnostic yield in these situations [34,37]. If the results continue to be indeterminate, the surgeon ultimately must decide whether to proceed with resection or terminate the case. This decision is a risk–benefit analysis weighing the likelihood of a benign resection against biopsy sampling error. The patient should be informed of this possibility preoperatively, and a decision should be made a priori about how to proceed in this scenario. Some patients may prefer a more aggressive approach, while others favor lung preservation in the absence of a cancer diagnosis.

### 7.2. Node Positive Disease Identified at Ebus

EBUS-TBNA may reveal unexpected nodal disease at the time of planned single anesthetic event lung cancer surgery. Further complicating matters is the uncertainty of reported diagnostic material on ROSE, without confirmatory pathology with immunohistochemistry stains. Similar to the discovery of occult disease at the time of pulmonary resection, in this scenario, the surgeon must decide whether to complete the resection and refer the patient for adjuvant therapy or stop and refer for neoadjuvant. While there is not a head-to-head trial comparing neoadjuvant regimens to adjuvant regimens with modern systemic therapy, guidelines recommend neoadjuvant treatment in the setting of node positive disease [9]. This recommendation stems from multiple randomized trials demonstrating excellent survival with an approach of neoadjuvant chemoimmunotherapy [5,7]. The PROSPECT-LUNG trial is actively investigating the question of whether a strategy of perioperative chemoimmunotherapy is superior to adjuvant alone [55]. In the scenario of occult disease discovered at the time of surgical resection, guidelines recommend proceeding with planned resection if a thoracotomy has been performed, but suggest stopping or continuing would be reasonable if the operation is minimally invasive [9]. In single anesthetic event lung cancer surgery, the decision should be an individualized approach weighing pathologic and patient factors. When in doubt, it is usually better to stop the procedure and convert to a staged approach. This gives an opportunity for tumor board discussion, and involvement of patient preferences into the clinical decision-making process. Success of the single anesthetic event pathway should not be defined by completion of resection within a single anesthetic, but by whether appropriate oncologic sequencing was preserved. Aborting the surgical portion of the procedure when unexpected nodal disease or other findings warrant neoadjuvant therapy or additional staging should be regarded as appropriate clinical judgment rather than a failure of the pathway; avoiding a second anesthetic must never take precedence over correct oncological decision-making.

### 7.3. Localization Fails

Failed localization during planned sublobar resection can be a significant challenge. Success rates for navigational and robotic bronchoscopy localization can range from 80 to 93%, making this a somewhat common scenario [48,56,57]. In this circumstance, the options include converting to an anatomic resection, or less commonly to reoperate with an alternative localization technique. If a non-anatomic wedge resection was planned, a segmentectomy can sometimes be performed as an alternative, but often a lobectomy would be necessary. The surgeon should ensure that the patient has adequate pulmonary reserve to tolerate a larger resection. It is important to include failed localization as part of the consent process to discuss the potential need for a more extensive resection due to this technical issue.

### 7.4. Frozen Section Is Discordant

Frozen section pathology provides the surgeon with key information about the diagnosis, margins and adequacy of resection. Occasionally the surgeon may encounter unexpected and discordant findings on frozen section pathology. For example, ROSE may be suspicious for malignancy, but the frozen section pathology reveals a benign diagnosis. In these circumstances, it is important to disclose the preliminary and discordant results to the patient and family, but also to wait for final pathology as additional stains can completely change the prognosis and conversation with the patient.

### 7.5. Equipment Failure

As with all emerging technologies, navigating equipment failure is a significant challenge in single anesthetic event lung cancer surgery. Given the sheer amount of equipment necessary to integrate video bronchoscopy, robotic bronchoscopy, on-table cone-beam CT, endobronchial ultrasound and VATS/RATS technology, equipment failure can occur somewhat commonly. Most issues can be solved through troubleshooting, and establishment of a specialized team who are familiar with the equipment can help. Occasionally, the decision must be made to either abort the procedure or convert to a staged approach if patient care cannot be safely delivered.

## 8. Limitations and Current Evidence Gaps

This manuscript is inherently limited as it is a narrative review. While the literature search represents both a search of common databases and an expert understanding of the key papers in the field, there may be omissions. At current, there are significant gaps in evidence for the single anesthetic event approach. There are no prospective trials comparing this approach to the standard-of-care-staged approach. There is no significant data reporting equivalent or improved survival from this approach. Many studies have short follow up, limiting conclusions on recurrence and survival. Nearly all published data in this space is retrospective. Most studies are single-center and limited to high-volume referral centers. Patient reported outcomes are largely unknown.

Cost-effectiveness is uncertain outside tertiary centers. There are significant barriers to implementation, both at the surgeon and hospital level. Long operative times can be difficult to justify hospital administration, despite clear benefits in total cost and overall procedural suite time. Compensation for each portion of the procedure is not always guaranteed. Lastly, there is a significant learning curve with the bronchoscopic portions of the procedure, and additional training is necessary to become facile with shape-sensing robotic bronchoscopy, cryobiopsy and endobronchial ultrasound. Perhaps the most significant barrier to widespread adoption is the concentration of resources required to deliver the complete pathway described in this review: robotic bronchoscopy, hybrid operating room and cone-beam CT capability, on-site cytopathology, thoracic surgical expertise, and a team experienced in transitioning efficiently between these components.

## 9. Future Directions

Future research in this space should work to minimize uncertainty and variability. Key areas for improvement are defining the optimal patients for single anesthetic event lung cancer surgery. The most immediate future priorities are prospective, multicenter validation of the single anesthetic event approach; standardized, reproducible patient-selection criteria; incorporation of patient-reported outcomes; formal cost-effectiveness analysis; and longer-term oncologic outcomes including recurrence and survival. Other areas for exploration include artificial intelligence (AI)-based tools. Emerging technologies may include deep learning informed inclusion and exclusion criteria, AI-augmented pathology, which could minimize variability, and novel techniques in lesion localization [58,59,60,61].

## 10. Conclusions

Single anesthetic event lung cancer surgery represents the integration of several technological advancements in minimally invasive thoracic surgery and bronchoscopy to streamline the diagnosis, staging and treatment of early-stage non-small-cell lung cancer. By combining shape-sensing robotic bronchoscopy, endobronchial ultrasound, biopsy, lesion localization and minimally invasive resection into a single anesthetic event, time from detection to resection can be significantly improved, while early retrospective series suggest acceptable perioperative safety in appropriately selected patients treated at experienced centers; prospective comparative data remain necessary to confirm oncologic and safety equivalence with traditional staged pathways.

## Figures and Tables

**Table 1:** Common clinical scenarios for single anesthetic event lung cancer surgery.

Clinical Scenario	Sequence of Interventions	Primary Indications & Lesion Characteristics	Strategic Rationale & Workflow Advantages
Comprehensive Diagnosis, Staging & Resection	Biopsy EBUS-TBNA Lobectomy	High clinical suspicion of early-stage NSCLC (solid nodules > 2 cm) requiring tissue diagnosis and mediastinal staging prior to definitive anatomic resection.	Streamlines the patient journey by avoiding sequential appointments. EBUS with ROSE provides negative invasive mediastinal staging (excluding N2/N3 disease within the limits of the nodes sampled) immediately prior to resection, preventing unnecessary lobectomies in cases of occult N2/N3 disease.
Complete Staging, Localization & Sublobar Resection	Marking EBUS-TBNA Sublobar Resection	Suspected early-stage peripheral < 2.0 cm NSCLC that require precise anatomical marking, coupled with a requirement for nodal staging.	Aligns with recent trial data (e.g., JCOG0802, CALGB 140503) supporting segmentectomy for small peripheral tumors. Ensures both clear surgical margins via marking and mandatory nodal evaluation in a single hybrid OR session.
Localization & Parenchymal-Sparing Resection	Bronchoscopic Marking Sublobar Resection	Subsolid nodules, ground-glass opacities (GGOs), or small/deep parenchymal lesions (<2 cm) with a high probability of malignancy.	Overcomes the inability to digitally palpate small or non-solid lesions during minimally invasive surgery (VATS/RATS). Modalities like ICG, dye, or microcoils ensure adequate margins during wedge or segmentectomy.
Concurrent Diagnosis, Localization & Limited Resection	Biopsy + Marking Sublobar Resection	Indeterminate solitary pulmonary nodules where pre-operative probability of malignancy is intermediate, and patients may have compromised pulmonary reserve.	Allows immediate tissue confirmation via intraoperative frozen section/ROSE. Simultaneous marking guarantees precise localization for an oncologically sound sublobar resection if the ROSE suggests NSCLC.

**Table 2:** Summary of outcomes from published literature.

Outcome	Number of Patients (*n*)	Published Range	Data Sources
Time Saved From Detection to Resection	*n* = 40	15 days [51]	Weiser et al. 2025 [51]
	*n* = 36	35 days [37]	Wolf et al. 2023 [52]
	*n* = 22	51 days [52]	Palleiko et al. 2025 [37]
Cost Savings	*n* = 36	Direct costs $2493, Total costs $3199 [37]	Palleiko et al. 2025 [37]
	*n* = 40	Direct costs $4524, Total costs $10,434 [51]	Weiser et al. 2025 [51]
Operative Time *	*n* = 65	218 min [42]	Perez et al. 2025 [42]
	*n* = 12	244 min [53]	Gordon et al. 2025 [53]
	*n* = 36	419 min [37]	Palleiko et al. 2025 [37]
Benign resection	*n* = 65	7.6% vs 21.9% w/standard of care [42]	Perez et al. 2025 [42]
	*n* = 36	16.7% [37]	Palleiko et al. 2025 [37]
Length of Stay	*n* = 12	1.8 days [53]	Gordon et al. 2025 [53]
	*n* = 40	3.5 days [51]	Weiser et al. 2025 [51]
	*n* = 36	3.6 days [52]	Wolf et al. 2023 [52]
Complications	*n* = 12–40	Air leak (0.0–8.3%) [51,53] Death (0%) [37,51,52,53] Any complication (0.0–39.0%) [37,51,52,53]	Weiser et al. 2025 [51], Gordon et al. 2025 [53], Pailleko et al. 2025 [37], Wolf et al. 2023 [52]

* Total operative time including all elements of the single stage procedure. The data presented in Table 2 are drawn from heterogeneous, non-randomized, single-institution series with differing patient selection criteria and outcome definitions. The data presented here should not be interpreted as pooled estimates.

## Data Availability

No new data were created or analyzed in this study. Data sharing is not applicable to this article.

## Glossary

AI	Artificial Intelligence
CT	Computed tomography
EBUS	Endobronchial ultrasound
EBUS-TBNA	Endobronchial ultrasound with transbronchial needle aspiration
ERAS	Early recovery after surgery
FDG PET/CT	Fluorodeoxyglucose positron emission tomography/computed tomography
FNA	Fine-needle aspiration
GGO	Ground-Glass Opacity
NSCLC	Non-Small-Cell Lung Cancer
RATS	Robotic-assisted thoracoscopic surgery
ROSE	Rapid on-site evaluation
SS-RAB	Shape-sensing robotic-assisted bronchoscopy
TTNB	Transthoracic needle biopsy
VATS	Video assisted thoracoscopic surgery
